# Direction-Specific Impairments in Cervical Range of Motion in Women with Chronic Neck Pain: Influence of Head Posture and Gravitationally Induced Torque

**DOI:** 10.1371/journal.pone.0170274

**Published:** 2017-01-18

**Authors:** Thomas Rudolfsson, Martin Björklund, Åsa Svedmark, Divya Srinivasan, Mats Djupsjöbacka

**Affiliations:** 1 Centre for Musculoskeletal Research, University of Gävle, Umeå, Sweden; 2 Department of Community Medicine and Rehabilitation, Physiotherapy, Umeå University, Umeå, Sweden; University of Bern, SWITZERLAND

## Abstract

**Background:**

Cervical range of motion (ROM) is commonly assessed in clinical practice and research. In a previous study we decomposed active cervical sagittal ROM into contributions from lower and upper levels of the cervical spine and found level- and direction-specific impairments in women with chronic non-specific neck pain. The present study aimed to validate these results and investigate if the specific impairments can be explained by the neutral posture (defining zero flexion/extension) or a movement strategy to avoid large gravitationally induced torques on the cervical spine.

**Methods:**

Kinematics of the head and thorax was assessed in sitting during maximal sagittal cervical flexion/extension (high torque condition) and maximal protraction (low torque condition) in 120 women with chronic non-specific neck pain and 40 controls. We derived the lower and upper cervical angles, and the head centre of mass (HCM), from a 3-segment kinematic model. Neutral head posture was assessed using a standardized procedure.

**Findings:**

Previous findings of level- and direction-specific impairments in neck pain were confirmed. Neutral head posture was equal between groups and did not explain the direction-specific impairments. The relative magnitude of group difference in HCM migration did not differ between high and low torques conditions, lending no support for our hypothesis that impairments in sagittal ROM are due to torque avoidance behaviour.

**Interpretation:**

The direction- and level-specific impairments in cervical sagittal ROM can be generalised to the population of women with non-specific neck pain. Further research is necessary to clarify if torque avoidance behaviour can explain the impairments.

## Introduction

Cervical function is important for activities of daily living involving stabilizing and orientating the head. Reduced cervical range of motion (ROM) is often reported in people with neck pain [1–7] and the magnitude of ROM impairment can be related to the level of pain and self-rated functioning [1, 7]. A better understanding of the characteristics of ROM impairments could help clarify the clinical relevance of these associations.

A majority of previous studies have measured cervical ROM in sagittal flexion/extension as a single joint head-thorax relationship. In a previous study we applied a simple kinematic method to decompose sagittal active cervical ROM into contributions from the upper and lower cervical levels separately [7]. We found that women with chronic non-specific neck pain had direction- and level-specific impairments compared to controls; reduced extension in the upper-, and reduced flexion in the lower cervical levels. In addition, we found that the lower levels contributed to a lesser extent to the total sagittal ROM in women with neck pain compared to controls. Those results suggest that a separation of upper and lower cervical contributions to sagittal ROM give more detailed information about impairments than traditional single-joint models and such information can allow for more specific treatment interventions. Here we aim to investigate two possible underlying causes behind the level and direction specific impairments.

A limitation in our previous study was that our procedure for obtaining a neutral sitting posture may not have been standardized properly. Since this position is used for defining the zero flexion/extension configurations, we could not rule out that the starting posture contributed to the direction-specific impairments. An improvement of the methodology would be to adopt a standardised procedure for obtaining starting posture (i.e., the zero head and neck configuration relative to laboratory-fixed coordinate system) and to anatomically anchor the entire kinematic model. This would allow for investigating if the direction-specific impairments could be attributed to differences in initial head posture between groups. Regarding the altered contribution to ROM between the cervical levels, we proposed that the impairments could reflect a strategy to minimize the torque exerted on the cervical spine [7]. This theoretical reasoning was based on ideas from Jull and co-workers (Chapter 14 in [8]) that impairments in control of cervical muscles may impair control of the head during global maximal flexion and extension. There is evidence that people with neck pain can have reduced strength in the deep cervical flexors [9, 10] and indications of similar impairments for the deep cervical extensor muscles [11]. Subjects with neck pain may strive to retain the centre of mass (CoM) of the head closer to the thorax to minimize the lever arm between the thorax and the gravitational force of the head. Predictions of such torque avoidance behaviour would imply that for tasks producing less torque on the cervical spine, the impairments would be of less magnitude compared to a high torque task such as global sagittal flexion/extension.

An alternate test of assessing cervical sagittal ROM is by head translation in protraction and retraction [12]. The postural aspects inherent in head translation are very different from those involved in global flexion/extension. As the head is kept in vertical alignment during the movement, the CoM migration is dependent on the ability to simultaneously flex and extend the upper and lower cervical levels. Given that head translation has been shown to maximize intervertebral ROM in the C0-C1 and C1-C2 levels compared to global flexion/extension [13], translation would allow for a greater intervertebral ROM while giving a smaller horizontal CoM migration compared to global flexion/extension. Notably, protraction involves flexion in the lower and extension in the upper levels of the cervical spine and this coincides with the greatest ROM impairments in neck pain reported in our previous study [7]. Thus, the combination of global flexion/extension and protraction could be used to investigate torque avoidance behaviour in people with neck pain.

In the present study, women with chronic non-specific neck pain and a control group performed active global cervical flexion/extension and head protraction. We aimed at gaining further insights into cervical ROM impairments reported by Rudolfsson and co-workers [7]. First, we hypothesised (i) that reduced extension in the upper- and reduced flexion in the lower cervical levels in people with neck pain can be explained by initial head posture. Second, we hypothesized (ii) that people with neck pain performing maximal cervical flexion/extension are limited by torque avoidance behaviour so that the difference in head CoM migration between neck pain subjects and healthy controls will be greater in maximal global flexion/extension than in maximal protraction.

## Methods

The study was part of the pre-intervention measurement of a randomized controlled trial (ISRCTN trial registration number, ISRCTN49348025). The design was cross-sectional with test type (global cervical flexion/extension and head protraction) as experimental condition. Written consent was given by all participants and the main intervention study was approved by the Ethical Review Board in Uppsala, Sweden.

### Participants

Participants with non-specific neck pain (NP) and healthy controls (CON) aged 20–65 years were recruited consecutively from august 2011 to march 2012. The age limit was set to include the working population in Sweden. The complete description of the recruitment is available in [14]. The NP participants fulfilled the inclusion criteria: neck region marked as their most dominant area of pain in a pain drawing; at least 6 weeks of pain duration; disability greater than “no disability” but less than “complete disability” assessed with the Neck Disability Index [15] and impaired productivity to work due to neck problems [16]. Exclusion criteria for the NP were: trauma related onset of neck pain, cervical rhizopathy, vestibular dysfunction, neurological, endocrinal, inflammatory or rheumatic disorder. Participants with fibromyalgia or temporomandibular disorders were also excluded. The participants in the control group also passed the above exclusion criteria.

### Data acquisition

We used an electromagnetic motion tracking system (FASTRAK, Polhemus Inc., Colchester, VT, USA) to measure the kinematics of the thorax and the head. Each receiver of the system enables the detection of six degrees of freedom (three coordinates and three orientation angles); therefore, the system could fully describe translation and rotation under rigid body assumptions. The transmitter of the system, defining the global coordinate system, was oriented so that the Y-Z plane was parallel to the sagittal plane of the subject [7]. A three-segment model of the thorax, cervical spine and head was constructed according to the method described in Rudolfsson et al. [7]. We improved the model by anchoring the entire model to anatomically defined locations to allow for group comparisons of natural posture. The inferior end of the thorax segment was anchored to the midpoint between Th8 and processus xiphoideus [17] and the kinematics of the segment was measured by a receiver attached to the dorsal spinal process of Th2. The cervical segment was defined as the vector between the Th2 receiver to the centre of rotation for the C0-1 joint, estimated from anatomical landmarks bilaterally of the mastoid processes. The head segment was defined from the C0-1 joint to the cantus of the eye. Kinematics of the head segment was measured by a second receiver on the forehead. The sampling rate was 40Hz.

### Test procedure

Since the task was not physically or cognitive demanding, we had no instructions on sleep or nutritional intakes at the day of testing. The participants were seated in a rigid chair with back support. To minimize thoracic movement belts were crossed over the chest and attached to the chair. Natural head posture was obtained by a self-balancing procedure in which the participants performed cervical flexion and extension movements with decreasing amplitude until a natural posture was obtained [18]. This procedure is most commonly used in standing but has shown good reliability in seated posture also [19]. The participants performed three repetitions of maximum global flexion/extension with the instruction: *bend your head forward/backward as far as possible*. The participants then performed three repetitions alternating between protraction and retraction with the instruction: *push your head forward/backward as far as possible*.

### Dependent variables

#### Direction-specific impairments of ROM

The angle between the thorax and cervical segments in the sagittal plane was used to define lower cervical (LC) flexion/extension and the angle between the cervical and head segment defined upper cervical (UC) flexion/extension. Flexion was defined as positive for both angles. The participants’ natural position after the standardised self-balancing procedure was used to define zero flexion/extension angles and zero horizontal lever arm between the head CoM (HCM) and the thorax. For hypothesis (i), maximum ROM measures for the global flexion/extension task was derived from the global head-trunk maximum, that is, when the sum of UC and LC angles had its maximum value for flexion or minimum value for extension. The dependent variables were upper cervical extension (UC_ext) and lower cervical flexion (LC_flex) in the global flexion/extension task. The covariates of posture were the UC and LC angles in the natural head position (UC_NHP and LC_NHP).

#### Torque avoidance

The origin of the coordinate system of the head was defined as the midpoint between landmarks of the external auditory meatus. We approximated the Frankfurt plane with a line between the origin and midpoint between the canthus of the eyes. The location of the HCM was assumed to be located 2.91 cm superior to the origin in perpendicular direction to the Frankfurt plane (table 16 in [20]) with kinematics measured by the head receiver. The gravitationally induced torque acting on the cervical spine during the ROM tasks was estimated by the horizontal distance between the HCM and the Th2 receiver to reduce influence of upper thoracic movement. HCM migration in global flexion/extension was determined separately for both directions of movement. For hypothesis (ii), the sum of the absolute values (HCM_global) was used as a dependent variable along with the HCM migration during protraction (HCM_prot).

**Fig 1:**
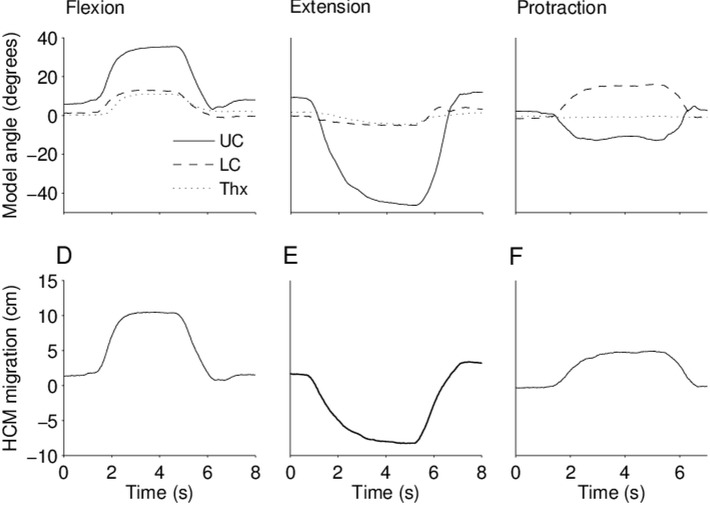
Upper (UC), lower (LC) and thorax segment (Thx) angles from the 3-segment model (upper panel), and head center of mass migration (HCM, lower panel) for one trial of global cervical flexion (left column), global cervical extension (middle column) and head protraction (right column) for a representative subject in the neck pain sample.

Kinematics for global flexion/extension and protraction are shown in Fig 1 for a representative NP subject. The time series of all trials were visually inspected independently by two authors (TR and ÅS) to ensure quality in the measurement data. Outcome angles and HCM migration from the measurement system were also validated against simultaneous photographs in the sagittal plane.

### Statistics

Participant characteristics were compared between groups with independent t-tests or Mann-Whitney U-test. Any group differences in age, length, BMI, weight or physical activity were considered as possible confounders in the analysis.

To validate our previous finding of direction-specific impairments in NP [7], we carried out two separate repeated measures ANCOVAs (RM ANCOVA) for the upper and lower cervical levels. Thus, ANCOVA of 2 (*group*: CON, NP) × 2 (*direction*: flexion—extension) design with within-subjects on the second factor and the corresponding covariate (UC_NHP and LC_NHP) were performed to test for a significant interaction between *group* and *direction*. To validate the previous finding of differential contribution to ROM between the upper and lower cervical levels in the two groups we calculated the ratio between ROM of the upper and lower cervical levels. These three analyses were performed both with and without possible confounders as covariates. The group effect size of the dependent variables between the adjusted and unadjusted analysis were compared. In the case of no major difference between the analyses, the possible confounder was left out in the following analyses.

#### Hypothesis (i)

To test hypothesis (i) that starting posture could explain the direction-specific (UC_ext, LC_flex) impairments in global flexion/extension in NP, we modelled the two dependent variables with both an ANOVA and an ANCOVA with the corresponding starting posture (UC_NHP and LC_NHP) separately as covariates. The covariates were checked for the assumption of homogeneity of the regression slopes and independence with the group factor. The resulting parameter estimates of group differences from the ANOVA/ANCOVA models with their 95% confidence intervals were used to compare the influence of starting posture on the dependent variables.

#### Hypothesis (ii)

To test the torque avoidance hypothesis (ii), we performed an RM ANOVA with factor *group* (NP, CON) and within subject factor *test* (HCM_global, HCM_prot). We rejected the null hypothesis if there was a significant interaction between *group* and *test*. To remove influence of starting posture in the protraction task, the HCM_prot was linearly detrended by using the residuals of a linear regression between LC_NHP and HCM_prot. Since the global flexion/extension task resulted in a much greater magnitude of HCM migration than the protraction task, we normalised HCM_global and HCM_prot by scalar multiplication to a mean value of 1 for the CON group. This was done to ensure that any statistical interaction was not due to the difference in magnitude of HCM migration between the two tests but only due to a relative reduction of group differences.

An important requirement of the protraction task is that the head is kept in horizontal alignment with the starting posture during the movement. This was tested by a RM ANOVA with factor *group* (CON, NP) and within-factor *horizontal_alignment* (start, stop) representing starting (equivalent to HE) and final sagittal projection of the angle between the head segment and the horizontal during the protraction task. An overall ability to perform the task was evaluated by the main effect of *horizontal_alignment* and differential group behavior by a significant interaction between *group* and *horizontal_alignment*.

#### Additional analyses

A data-driven analysis was performed to test the within-subject association between HCM migration in the protraction and global flexion/extension tasks with a Pearson product-moment correlation for each group. Correlation was also used to determine the association between UC_NHP and UC_ext, and between LC_NHP and LC_flex, respectively for all participants.

All statistical tests were carried out with IBM SPSS Statistics version 22 and we used a significance level of 0.05 throughout all analyses. The residuals of all models were checked for possible violation of the assumption of normal distribution.

### Sample size calculations

Unpublished test-retest data from 13 healthy controls was used to construct a nested random-effects model for each outcome to partition the total variance into between-subjects and within-subjects components (Table 1). We estimated the symmetrical sample sizes required to detect a difference of 20% in HCM_global and HCM_prot between healthy controls and people with neck pain in an independent t-test at a significance level of 0.05 and a statistical power of 0.8. The size of the minimum group difference of interest was based on findings in Rudolfsson et al. [7].

**Table 1:** The between- and within-subject variance components of ROM and postural variables calculated from an unpublished test-retest reliability study (N = 13). The required size of each group was estimated for 20% detectable difference between groups for the HCM_global and HCM_prot.

Dependent variable	Between-Subjects variance [95% CI]	Within-Subjects variance [95% CI]	Required number of subjects per group
HCM_global (cm)	4.48 [1.23; 13.99]	0.92 [0.47; 2.49]	7
HCM_prot (cm)	1.54 [0.09;4.94]	0.67[0.35; 1.75]	43

CI: Confidence interval; HCM_global: The sum of the absolute values of head center of mass migration in global flexion and extension; HCM_prot: Head center of mass migration during protraction.

## Results

Descriptive statistics of the participants’ characteristics are presented in Table 2. The shortest duration of pain reported by participants was 3 months. All participants were able to complete both tests. For the global flexion/extension test, all 960 trials were included in the data processing. For the protraction test, four trials out of 480 were discarded due to aberrant movements. Descriptive statistics for all kinematic variables are presented in Table 3.

**Table 2:** Descriptive statistics for the control and neck pain groups (mean and SD except for duration that is median and inter quartile range). The NDI is normalised to the range of 0 to 100.

Characteristics	CON (n = 40)	NP (n = 120)	p-value
Age (years)	46,9 (11,8)	47,3 (11,6)	0,85
BMI	23,3 (2,8)	24,7 (4,2)	0,09
Weight (kg)	65,9 (8,8)	67,4 (11,8)	0,30
Height (cm)	167,2 (5,6)	165,8 (5,6)	0,19
Physical activity (1–6)	5,0 (0,8)	4,3 (1,0)	<0,01
SF-36 PCS	55,9 (3,6)	41,7 (6,8)	<0,01
SF-36 MCS	54,2 (7)	49,8 (8,5)	0,09
Duration (months)	N/A	60 (24–124)	N/A
NRS pain	N/A	4,62 (1,8)	N/A
NDI	N/A	23,2 (8,8)	N/A

CON: Control group; NP: Neck pain group; BMI: Body mass index; SF-36 PCS: Short Form 36 physical component summary; SF-36 MCS: Short Form 36 mental component summary; Physical activity: How physically active at leisure time have you been in the last year? (1–6); NRS pain: Numerical rating scale of pain (0–10); NDI: Neck Disability Index; N/A: not applicable.

**Table 3:** Descriptive statistics for all kinematic variables separately for the control and neck pain groups (Mean (SD)). All variables have unit degrees except for the HCM variables that have unit cm.

Variable	CON	NP
UC flex	36.3 (7.8)	33.7 (7.0)
UC ext	-53.3 (9.9)	-46.0 (10.6)
LC flex	16.3 (5.3)	11.8 (6.0)
LC ext	-2.6 (5.9)	-1.8 (4.7)
HCM_flex	9.7 (1.5)	8.2 (1.9)
HCM_ext	-10.2 (2.5)	-7.9 (2.7)
HCM_global	19.9 (3.2)	16,2 (3.8)
HCM_prot	5.2 (2.0)	4.3 (1.5)
UC_NHP	9.5 (12.2)	10.6 (9.8)
LC_NHP	68.4 (7.4)	70.1 (7.5)

CON: control group; NP: neck pain group; UC_flex; Upper cervical flexion in global flexion; UC_ext; Upper cervical extension in global extension; LC_flex; Lower cervical flexion in global flexion; LC_ext; Lower cervical extension in global extension; HCM_flex; Head center of mass horizontal migration in global flexion; HCM_ext; Head center of mass horizontal migration in global extension; HCM_global: Total range of head center of mass horizontal migration in global extension and extension; HCM_prot: Head center of mass horizontal migration during protraction; UC_NHP: upper cervical angle in the natural head position; LC_NHP: lower cervical angle in the natural head position.

**Fig 2:**
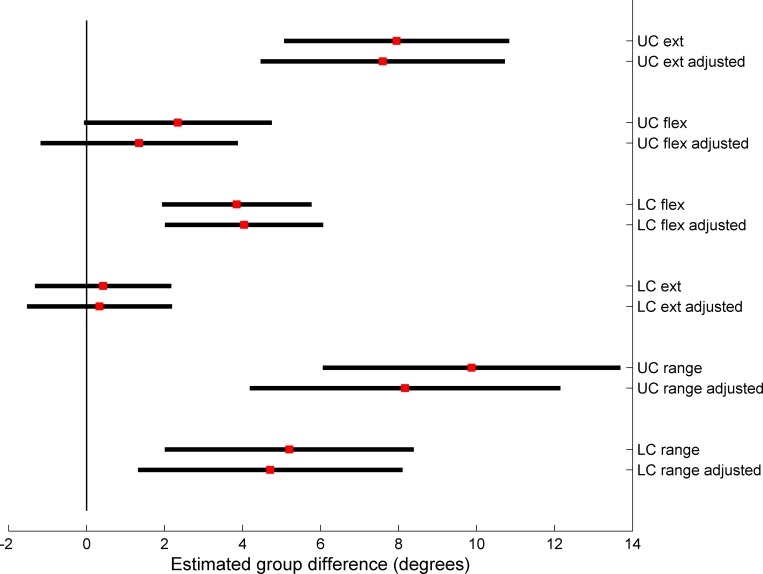
Forest plot of parameter estimates of group differences between neck pain participants and controls along with 95% confidence intervals for adjusted (ANCOVA) and unadjusted (ANOVA) analyses of cervical range of motion (ROM). The covariate was self-rated physical activity. Positive values favour increased ROM in the control group compared to the neck pain participants.

The ANCOVAs of ROM in global flexion-extension showed significant interaction for *direction × group* for both the upper (p = 0.006) and lower (p = 0.004) cervical levels as well as significant main effects of group. The significant interactions supports that the ROM impairments in NP compared to CON are direction-specific with greater impairments in extension in the upper levels and flexion in the lower levels. In addition, there was a significant difference in the contribution of ROM between the upper and lower cervical levels between groups (p = 0.02) and the UC/LC ratio was greater in NP compared to CON. Among the potential confounders, there was significant group difference in physical activity (Table 2) and the aforementioned analyses were also performed with physical activity as a covariate. As seen in Fig 2, the effect on the dependent variables and the confidence interval limits were negligible, and in addition the covariates were not significant in the models. Based on this, the covariate was not used in the following analyses.

### Direction-specific impairments of ROM

**Fig 3:**
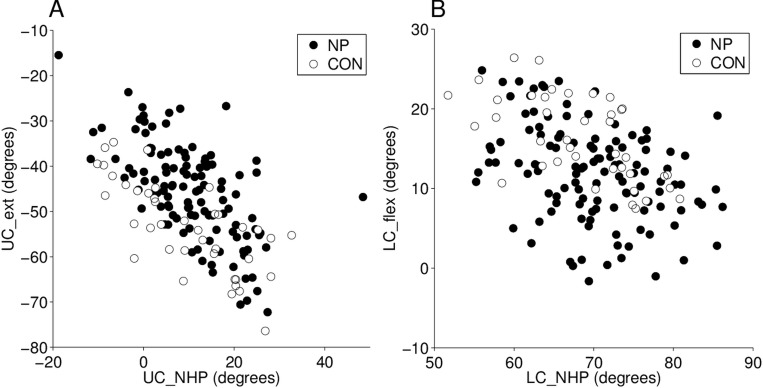
Scatterplot of the upper cervical angle in the natural head posture (UC_NHP) versus upper cervical ROM in extension (UC_ext) in A, and the lower cervical angle in the natural head posture (LC_NHP) versus lower cervical ROM in flexion (LC_flex) in B. Filled circles represent the neck pain subjects (n = 120) and open circles represent controls (n = 40).

Fig 3 illustrates the relationships between initial head posture and upper cervical ROM in extension and lower cervical ROM in flexion separately. Correlation coefficients were r = -0.60 for UC_NHP vs. UC_ext and r = -0.43 for LC_NHP vs. LC_flex.

To test hypothesis (i) that the direction-specific ROM impairments in NP can be explained by starting posture we first tested if starting posture (UC_NHP and LC_NHP) were different between groups. The t-tests showed no significant group differences for neither UC_NHP nor LC_NHP (p = 0.57 and p = 0.21 respectively). Then we modelled the group difference for UC_ext and LC_flex with both an ANOVA and an ANCOVA (UC_NHP as covariate in the model for UC_ext and LC_NHP as covariate in the model for LC_flex).

**Fig 4:**
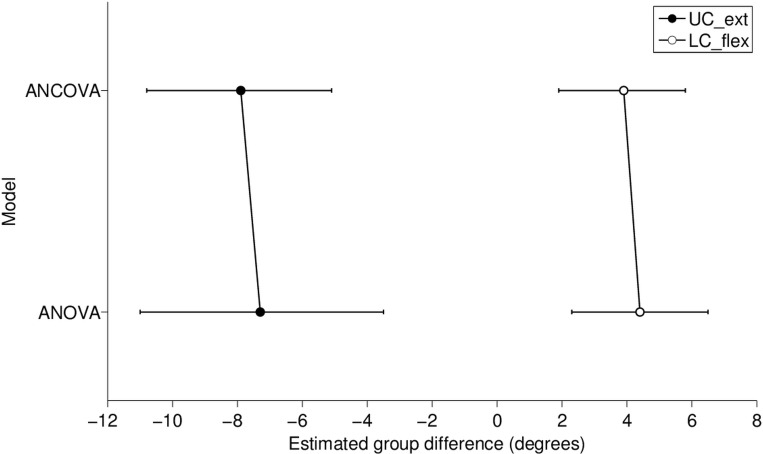
Parameter estimates of group differences in degrees along with 95% confidence intervals separately for upper cervical extension (UC_ext) and lower cervical flexion (LC_flex) for the models with (ANCOVA) and without (ANOVA) initial posture as covariate. For UC_ext, a more negative value represents greater impairment in the NP group compared to CON. For LC_flex, a more positive value represents a greater impairment in the NP group compared to CON.

As illustrated in Fig 4, for UC_ext the group difference and confidence interval were -7.3 [-11.0; -3.5] for the ANOVA and -7.9 [-10.8; -5.1] for the ANCOVA. The corresponding estimate for LC_flex was 4.4 [2.3; 6.5] for the ANOVA and 3.9 [1.9; 5.8] for the ANCOVA.

The non-significant group differences for the initial postures and only minor changes in estimates of group difference between ANOVA and ANCOVA models (in relation to the confidence intervals of the estimates) does not support our hypothesis (i); i.e., that the direction-specific ROM impairments can be explained by starting posture.

### Torque avoidance

**Fig 5:**
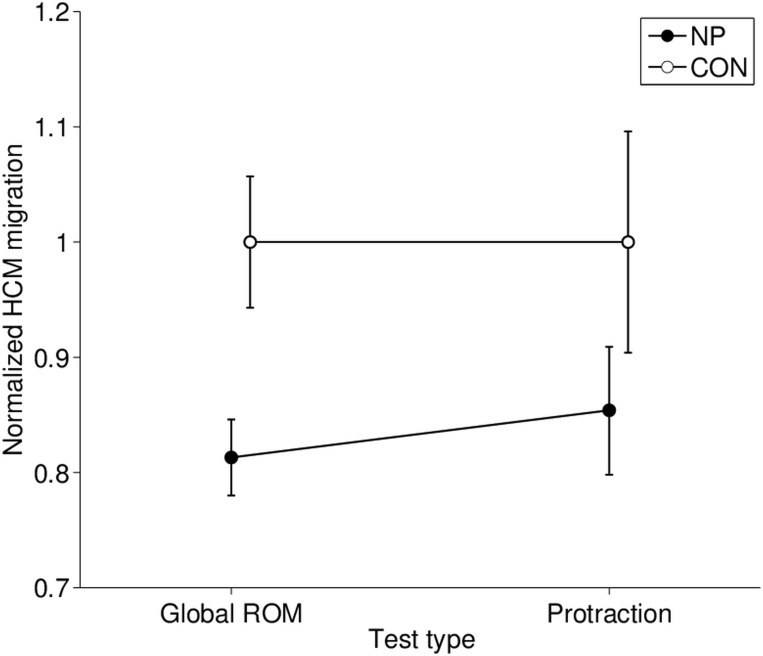
Interaction plot *group × test* for HCM migration normalised to the averages for the CON group separately for each test, with 95% confidence intervals.

The ability to perform the protraction task with the head in horizontal alignment with the starting posture during the movement was evaluated with a RM ANOVA (within subject factor *horizontal_alignment* and between subject factor *group*). The non-significant main effect of *horizontal_alignment* (F(1, 158) = 2.79, p = 0.10) support the conclusion that the overall ability to perform unguided protraction was sufficient with only slightly increased flexion (mean = 0.98, SD = 7.54 degrees) at maximal protraction. There was no differential group behaviour (*group × horizontal_alignment*, F(1, 158) = 0.25, p = 0.62). Descriptive data for HCM migration in the two tasks are presented in Table 3. For hypothesis (ii), the RM ANOVA showed a significant main effect of *group* (F(1, 158) = 21.57, p<0.001) and a non-significant interaction for *group × test* (F(1, 158) = 0.50, p = 0.48), also illustrated in Fig 5. The non-significant interaction lends no support for hypothesis (ii).

### Additional analyses

The within subject association between HCM migration in the protraction and global flexion/extension tests showed low correlations; r = 0.24 for NP and r = 0.25 for CON, corresponding to an explained variance of only 6%.

## Discussion

The present study confirmed our previous findings of direction- and level-specific impairments in ROM in active sagittal cervical flexion/extension in women with non-specific neck pain. The main aim of our study was to investigate possible underlying causes for these impairments. We hypothesised (i) that the initial posture defining zero flexion/extension, and (ii) that a behavioural strategy to avoid large torques on the cervical spine, could explain the direction- and level-specific impairments. The results did not support these hypotheses.

Comparing the present results to the results from our previous study showed that the magnitude of global ROM (Table 3) is similar to our previous findings [7] for both the CON and NP groups. The significant interaction between group and direction confirmed that the direction-specific impairments in cervical ROM were also present in the current sample. The lesser contribution to global ROM from the lower cervical levels in NP compared to CON in our previous results was also confirmed in the present sample. These confirmations of previous findings support that the direction- and level-specific impairments in ROM for active sagittal cervical flexion/extension can be generalised to the population of women with non-specific neck pain. These results also constitute a solid foundation for testing the hypotheses of the present study.

We used a procedure of self-balancing the head into a natural head posture [18] to define zero flexion/extension and fully anchored our kinematic model to anatomical landmarks. Contrary to our hypothesis (i), the results showed non-significant group differences in the posture estimates from the three-segment model and the magnitude of group difference in UC_ext and LC_flex was not altered by adding the posture estimates (UC_NHP and LC_NHP) as covariates.

Notably, from the correlation analysis of the relation between ROM-estimates and initial postures, the unexplained variance was greater for the relationship between LC_NHP and LC_flex than for UC_NHP and UC_ext (also evident from Fig 3). Since the thorax is not a rigid body, there is a risk for a slight change in flexion/extension of the thoracic spine between the collection of anatomical landmarks for the thorax segment (performed before the start of the test) and the recording of the normal posture. This may introduce slight errors in the anchoring of the inferior end of the thorax segment. It is possible that the greater unexplained variance for the relationship between LC_NHP and LC_flex is related to this methodological issue. However, we consider it unlikely that this source of variability would introduce bias between groups or obscure underlying group differences in posture because the mean posture estimates were very similar between groups. Hence, we conclude that the direction-specific impairments cannot be explained by starting posture.

To test hypothesis (ii) we compared the relative magnitudes of HCM horizontal migration in maximal global flexion/extension and in maximal protraction between groups. The non-significant interaction between the factors *test* and *group* supports that the impairment in HCM migration in the NP group relative to the CON group were of equal magnitude in the two tests. This finding does not support that active ROM in global flexion/extension in NP is reduced due to a strategy to avoid high torque in the cervical spine.

For both groups, the magnitude of HCM migration in global flexion as well as in global extension was about 2 times greater than in protraction (Table 2). This supports that the gravitationally induced torque on the cervical spine was greater in both directions of the global flexion/extension test compared to protraction since the HCM migration magnitude is proportional to the leaver arm between the thorax and the gravitational pull on the head. Thus, the two tests most likely provided a clear contrast in torque magnitude required for testing hypothesis (ii).

The movement in the protraction task requires keeping the head in horizontal alignment throughout the movement. This will likely pose greater challenge on muscle coordination compared to global flexion/extension, since it requires flexion in the lower segments with simultaneously extension in the upper segments, in contrast to global activation of flexors or extensors. Protraction may thus require more elaborate coordination between the deep and the superficial cervical muscles, the lower and upper regions of the deep muscles, and also pose high demands on precise directional specificity of muscle activation to avoid co-contraction of movement antagonists. Impairments in these motor functions have been reported for people with neck pain [9, 21, 22]. The added complexity of inter-segmental coordination intrinsic to the protraction task may thus be a factor behind the impairments in the protraction task and may therefore have obscured any effects of torque avoidance behaviour.

Based on the above reasoning, together with the negative results from the test of hypothesis (ii) and the fact that both HCM_global and HCM_prot separate groups distinctly (evident from Fig 4), it may be argued that two different mechanisms underlie the impairments. To shed light on this possibility we tested the within-subject association between HCM migration in the protraction and global flexion/extension tests. This revealed very low correlations corresponding to an explained variance of only 6%. Thus, HCM migration magnitude for the two tests seems to reflect different underlying mechanisms.

We proposed the two hypotheses as tentative explanations of our previous findings of cervical ROM impairments in women with neck pain [7]. The hypotheses were not contradictory but could have been present in parallel, however, they were based on results from a single sample. This may have called for alpha correction not to increase the family-wise error rate. Notably though, as a first step in the present study, we verified our previous results in our current sample. This verification was a pre-requirement to ascertain that our hypotheses were based on repeatable results. The results of the present study suggested rejection of both hypotheses. The use of a protraction task as a low torque contrast to global flexion/extension can be questioned since protraction may put greater demands on muscle coordination than global flexion/extension, thus obscuring possible effects of a torque difference. The low correlation between the tests lends support to this notion. Future research should strive to investigate tasks with different magnitude of torque but with similar coordinative complexity.

The sample size was pre-determined by the design from a longitudinal study, thus we could only verify if the sample size was adequate for this cross-sectional study. The sample size calculation showed that the protraction task required 43 participants for each groups in a symmetrical allocation ratio. Since the longitudinal study included 120 and 40 participants per group, we feel confident that the oversampling in the neck pain group provided observations to ensure approximately 80% power in the study.

A delimitation of our study was to investigate only movement in the sagittal plane. This consideration was based on the fact that flexion/extension of the neck is a risk factor for developing neck pain [23] and that the three-segment model used for decomposing cervical ROM cannot be applied to axial rotations.

## Conclusions

In the present study we reproduced our previous findings of direction- and level-specific impairments in ROM for active cervical sagittal flexion/extension in women with non-specific neck pain. This supports that the findings are valid and that the direction- and level-specific impairments can be generalised to the population of women with non-specific neck pain. Therefore, these results are valid to consider by physiotherapists in clinical evaluations. We tested two hypotheses as explanations of the direction- and level-specific impairments. We conclude that the impairments cannot be explained by starting posture. The results did not support that active ROM in global flexion/extension is reduced due to a strategy to avoid high torque in the cervical spine. However, the complexity of inter-segmental coordination intrinsic to the protraction task used as low torque condition may have obscured a test contrast related to torque differences. Thus, further research is needed to fully reject the torque avoidance hypothesis.

## Supporting Information

S1 Dataset CROM Datafile.xlsx. (XLSX)
